# Maternal Diet and Morbidity Factors Associated with Low Birth Weight in Haiti: A Case–Control Study

**DOI:** 10.1089/heq.2017.0063

**Published:** 2018-07-01

**Authors:** Abdirahim Rashid, Thomas Park, Kenneth Macneal, Lora Iannotti, Will Ross

**Affiliations:** ^1^ Barnes-Jewish Hospital, Washington University School of Medicine, St. Louis, Missouri.; ^2^ University of Pennsylvania Health System, Philadelphia, Pennsylvania.; ^3^ Brown School at Washington University in St. Louis, St. Louis, Missouri.

**Keywords:** developmental origins of health and disease, dietary diversity, Haiti, hypertension, low birth weight

## Abstract

**Objective:** A matched, case–control study was conducted to examine the association between development of low birth weight (LBW) and maternal factors, including dietary intake, comorbidities, and socioeconomic factors, among women in Cap Haitien, Haiti.

**Design:** Mothers who delivered LBW babies; defined as ≤2.5 kg, were identified by review of the medical record and matched to mothers of similar age, parity, with normal birth weight (NBW) babies. A survey was administered consisting of Women's Dietary Diversity Score (WDDS), maternal reporting of comorbidities, income, and educational level.

**Subjects:** Women were eligible if they delivered and had newborns weighed within the last 2 years. Total study participants consisted of 32 cases and 34 controls matched for age, parity, and month of delivery.

**Results:** Mothers who consume eggs were 78% less likely to have given birth to a LBW infant (OR 0.22 (95% CI: 0.05–0.87). Mothers with NBW babies had a nonsignificant trend towards higher WDDS. The prevalence of hypertension in mothers who were seen in the clinic at least once over the past 2 years was found to be 27%, and 78% of mothers were not aware of their diagnosis.

**Conclusion:** Enhancing maternal nutrition during pregnancy has broad implications for reducing LBW, improving fetal health and reducing fetal predilection for chronic diseases in adulthood. Longitudinal prospective studies are needed to evaluate the selective benefit of eggs and other high-quality foods in protecting fetal growth. Efforts to improve knowledge and awareness of hypertension in Haiti should be undertaken.

## Introduction

According to the Developmental Origins of Health and Disease Hypothesis, maternal micronutrient insufficiency leads to fetal undernutrition, causing maladaptive changes and increased risk of chronic diseases in adulthood.^1–3^ Low birth weight (LBW) is associated with increased risk of coronary artery disease and hypertension in old age.^3–8^ Characterizing the impact of being born LBW on noncommunicable diseases in developing countries is challenging due to paucity of maternal health data and birth weight records. In Haiti, it is estimated that 21% of children are born LBW, defined as weighing at or below 2.5 kg.^9^ Among the factors contributing to LBW, lack of dietary diversity with few fruits, vegetables, and animal sources of food has been noted in low and middle-income countries.^10^ Maternal hypertension was also noted to be a significant risk factor for LBW infants in recent Haitian immigrants to the United States.^11^

Haiti is one of the poorest countries globally, ranking 168 out of 187 countries on the Human Development Index.^12^ In Haiti, 14% of school age children are stunted at baseline, another 14.5% are underweight, while 65% of children aged 6–59 months are anemic.^13,14^ Low socioeconomic status and poverty play a role in limiting food in pregnant women.^15,16^ Poor gestational nutrition, low prepregnancy weight, short maternal stature, general morbidity, and general episodic illness are known major determinants of intrauterine growth restriction and subsequent LBW.^17^ In contrast, increased intake of green leafy vegetables, fruits, and milk products have been associated with increase in birth weight.^18^

We examined the association between maternal dietary diversity, anemia, blood pressure, and reporting of comorbidities such as diabetes, hypertension, and socioeconomic status on the development of LBW infants in a matched, case–control study conducted in Cap-Haïtien, Haiti. To determine the potential for future medical interventions, we also examined knowledge and awareness of hypertension by administered a questionnaire. Dietary diversity was used as a proxy for nutrient adequacy by tabulating a Women's Dietary Diversity Score (WDDS). The Women's Dietary Diversity Project validated WDDS as an instrument to predict micronutrient adequacy in females of reproductive age in a number of diverse settings.^10^ As a response, a WDDS consisting of nine food groups was adopted by the Food and Agriculture Organization of the United Nations (FAO). A score of 4 or more has been shown to be associated with lower risk of maternal anemia and giving birth to LBW infants.^19^ The use of dietary recall to reflect the dietary habits of a previous pregnancy has been validated in a previous study.^20^ At the time of submission, to the best of our knowledge, there have been no clinical studies addressing dietary diversity, hypertension awareness, and birth outcomes in Haiti.

## Methods and Materials

### Participants

This study was conducted according to the guidelines laid down in the Declaration of Helsinki and all procedures involving human subjects/patients were approved by the Institutional Review Board (IRB) at Washington University in St. Louis as well as Haitian IRB through the Ministry of Health of Haiti. Written informed consent was obtained from all subjects/patient with the assistance of an interpreter. The study was conducted in Cap-Haitien, Haiti from March to April 2016. All records were obtained from Hospital Fort St. Michel, a public clinic in an urban community in Cap Haitien. Medical records were selected at random from all patients at the hospital who were seen between September 2014 and February 2016. Records were screened for obstetric visits where a birth weight was recorded. Over 90% of the mothers of LBW infants were called using telephone numbers in the hospital records. The affiliation of the authors, an outline of the study, and a small remuneration for participation ($5 USD) was communicated to potential participants. Each mother of a LBW infant who agreed to participate was then matched by age at delivery, parity, date of infant's birth, and gestational age to 1–2 mothers of infants with normal birth weight (NBW). These mothers were contacted using the same process outlined above.

### Data collection

All study components were conducted in Haitian Creole by local nurses who were hired and trained by the study authors. The study was conducted in a dedicated room within Hospital Fort St. Michel. Patient consent was obtained upon arrival, after which the survey was administered, and finally anthropometric data were collected.

Information on food consumption was collected by a qualitative dietary recall over the previous 24 h, based on the WDDS. The women were asked to recall all the dishes, sauces, snacks, drinks, and other foods they had consumed the previous day. The women were then prompted for any additional foods and asked to describe all the ingredients in the food they had eaten. The ingredients were subsequently translated into English and coded into a predefined list of 10 food groups: starchy staples (cereals/roots/tubers); dark green leafy vegetables; vitamin-A-rich fruits and vegetables; other fruits and vegetables; organ meat; flesh meat and fish; eggs; milk and dairy products; legumes and nuts; and oils and fats. This information allowed the construction of WDDS. They were then asked to complete a multiple-choice survey containing questions about hypertension.

Hemoglobin concentrations were measured using the HemoCue system and reported in grams per 100 milliliters (g/dL). The HemoCue system was cleaned and calibrated each morning before sample collection. Blood was collected with a fingerstick and a microcuvette in one continuous process and tested in the HemoCue system. For anthropometry, the Seca Model 874 (Digital) 440 lb×0.1 lb resolution scale and the ShorrBoard height measuring board were used to collect weight (to the nearest 0.1 kg) and height measures (to the nearest 1 mm), respectively, using international protocols. We also recorded the vital signs, including blood pressure with a manual sphygmomanometer, for all the participants.

### Statistical analyses

Characteristics of mothers with and without LBW infants were compared using the chi-square test for categorical variables and Student's unpaired *t* test for continuous variables. A *p* value of ≤0.05 was accepted as significant. Odds ratios were calculated with each food group being the exposure variable and LBW the outcome variable. All analyses were performed using the statistical software package SPSS (Version 23).

## Results

Records of more than 15,000 clinic visits between September 2014 and February 2016 were reviewed. Of these, 1046 were records of deliveries where a birth weight was documented. There appeared to be no seasonal variation in birth weight (data not shown). Of all the birth records reviewed, 172 (16%) of these births were of infants ≤2.5 kg. Fifty-six (33%) of the infants gestational ages were not documented in the chart, but of those that were documented, 94 were full term (≥37 weeks). One hundred fifty-three mothers of LBW infants were called to participate in the study. Majority of study participant's telephone numbers obtained from the chart were disconnected and could not be reached. However, of those whose numbers were still in service, greater than 90% were able to partake in the study. A total of 32 mothers of LBW infants and 34 mothers of NBW infants completed the survey and anthropometric data collection.

Outside of infant birth weight, there were no significant differences across the pregnancy-related, anthropometric, or socioeconomic data collected between the case and control groups (Table 1). The average birth weight for mothers of infants with LBW was 2.09 kg (SD 0.41), compared with 3.08 kg (SD 0.33) in the control group (*p*<0.001). There was no significant difference in WDDS between the cases and controls when measured as a mean (5.5±1.52 in the cases vs. 5.79±1.30 in the controls, *p*=0.40) or as a dichotomous variable for presence of a diverse diet, meaning ≥5 food groups (28% vs. 18% in cases and controls, respectively, *p*=0.31) (Table 2). Subgroup analysis by food group showed mothers who consume eggs were 78% less likely to have given birth to LBW infants (OR 0.22; 95% CI: 0.05–0.87) (Table 2).

**Table 1.** **Characteristics of Mothers in the Case (with Low Birth Weight Infants) and Control Groups (with Normal Birth Weight Infants)**

	Case group (n=32)	Control group (n=34)	p
Pregnancy-related parameters			
Maternal age at birth, mean and SD	27.63±6.3	27.6±5.5	0.95
<20	3 (9%)	2 (6%)	0.98
20–29	20 (63%)	20 (59%)	
30–39	8 (25%)	11 (32%)	
>40	1 (3%)	1 (3%)	
Gestational age, mean and SD	38.8±3.0	39.3±1.7	0.40
<37 weeks	2 (6%)	2 (6%)	0.87
37–41 weeks	19 (59%)	14 (41%)	
>=41 weeks	3 (9%)	6 (18%)	
Unknown	8 (25%)	12 (35%)	
Previous pregnancies, mean and SD	0.94±1.4	1.0±1.2	0.85
None previous	17 (53%)	14 (41%)	0.84
1–2 previous	12 (38%)	17 (50%)	
>2 previous	3 (8%)	3 (9%)	
Birth weight, mean and SD	2.09±0.41	3.08±0.33	<0.001^*^
Anthropometric data at time of study			
BMI, mean and SD	22.6±3.6	23.4±4.4	0.42
Underweight (BMI <18.5)	3 (9%)	5 (15%)	1.00
Healthy (BMI 18.5–24.9)	21 (66%)	18 (53%)	
Overweight (BMI 25.0–29.9)	7 (22%)	8 (24%)	
Obese (BMI >30.0)	1 (3%)	3 (9%)	
Systolic blood pressure, mean and SD	115±22	118±18	0.55
Diastolic blood pressure, mean and SD	75±13	80±15	0.15
Heart rate, mean and SD	83±15	83±10	1.00
Hemoglobin, mean and SD	12.5±1.7	12.0±1.5	0.21
Socioeconomic data at time of study			
Monthly income			0.78
<100 Haitian dollars	5 (15%)	4 (12%)	
100–500 Haitian dollars (US$7–36)	8 (24%)	10 (30%)	
501–800 Haitian dollars (US$36–58)	1 (3%)	6 (18%)	
801–1000 Haitian dollars (US$58–72)	3 (9%)	2 (6%)	
>1000 Haitian dollars (US$72)	15 (44%)	11 (33%)	
Missing	0	1	
Education level			0.97
No education	1 (3%)	1 (3%)	
Primary school	10 (31%)	13 (39%)	
Secondary school	18 (56%)	18 (55%)	
University/graduate school	3 (9%)	1 (3%)	
Missing	0	1	

^*^ Statistically significant at *p*<0.05.

LBW, low birth weight.

**Table 2.** **Women's Dietary Diversity Survey Results in Mothers with Low Birth Weight (Case) and Normal Birth Weight (Control) Infants**

	Case group (n=32)	Control group (n=34)		
	n (%)	n (%)	OR (95% CI)	p
Food group, *n* and % of total				
Starchy staples	32 (100)	34 (100)	N/A	1.00
Dark green leafy vegetables	15 (47)	14 (41)	1.26 (0.48–3.34)	0.64
Vitamin-A rich fruits and vegetables	19 (59)	18 (53)	1.30 (0.49–3.45)	0.60
Other fruits and vegetables	25 (78)	26 (76)	1.10 (0.35–3.48)	0.87
Organ meat	1 (3)	3 (9)	0.33 (0.03–3.38)	0.33
Meat and fish	31 (97)	32 (94)	1.94 (0.17–22.47)	0.59
Eggs	3 (9)	11 (32)	0.22^*^ (0.05–0.87)	0.022^*^
Legumes and nuts	25 (78)	28 (82)	0.77 (0.23–2.58)	0.67
Milk and milk products	13 (41)	17 (50)	0.68 (0.32–2.31)	0.45
Oils and fats	12 (38)	14 (41)	0.86 (0.32–2.31)	0.76
Average WDD^a^ score, mean and SD	5.5±1.52	5.79±1.30		0.40
WDDS <5, *n* and% of total	9 (28)	6 (18)	1.83 (0.57–5.89)	0.31

^*^ Statistically significant at *p*<0.05.

WDDS, Women's Dietary Diversity Score.

In a subanalysis of hypertension knowledge and awareness among mothers screened during the study, none of the 66 were able to correctly define hypertension (Table 3). Only 21% could identify the association between hypertension and stroke, heart disease, and kidney disease (Table 3). The prevalence of hypertension in this study (27%) is similar to that previously reported for this group of young females.^21^ Only 22% reported having high blood pressure, and only 33% had been told by their doctor that their blood pressure needed to be controlled. Out of the entire sample, less than half (41%) believed that they could afford medications to treat hypertension.

**Table 3.** **Hypertension Knowledge and Awareness by Blood Pressure on Day of Survey**

	Normotensive, (n=48)	Hypertensive, (n=18)		Entire sample, (n=66)
	n (%)	n (%)	p	n (%)
Correctly defined hypertension	0 (0)	0 (0)	1.00	0 (0)
Reports current high blood pressure	2 (4)	4 (22)	0.02^*^	6 (9)
Ever been told by a doctor that their blood pressure needs to be controlled	12 (25)	6 (33)	0.50	18 (27)
Believes that high blood pressure needs to be treated	41 (85)	12 (67)	0.09	53 (80)
Believes that they can afford medications	20 (42)	7 (39)	0.84	27 (41)
Correctly identified hypertension as a cause of stroke, heart disease, and kidney disease	11 (23)	3 (17)	0.58	14 (21)

^*^ Statistically significant at *p*<0.05.

## Discussion

In this matched case–control study, we aimed to characterize LBW in Haiti by looking at a number of factors known to contribute to the development of LBW infants. Mothers who consumed eggs were significantly less likely to have given birth to a LBW baby. Comparison of the dietary habits, socioeconomic standing, hemoglobin concentration, blood pressure, BMI, and maternal reporting of comorbidities between mothers with and without LBW babies did not reveal any significant difference. A separate questionnaire assessing knowledge of hypertension and awareness found profound poor comprehension and lack of awareness. The majority of study participants did not know the impact of untreated hypertension or have the financial means to afford prescription medications to treat high blood pressure.

There is emerging literature supporting the benefits of egg consumption during pregnancy.^22–24^ In a randomized control trial conducted in Ecuador,^22^ children ages 6–9 months were assigned to an intervention of one egg per day for 6 months. The investigators noted a reduced prevalence of stunting by 47% (prevalence ratio [PR], 0.53; 95% CI: 0.37–0.77) and underweight by 74% (PR, 0.26; 95% CI: 0.10–0.70) in the children who consumed eggs. Eggs are commonly available, relatively inexpensive, and contain nutrients that may be of benefit during pregnancy as well as during weaning, including vitamin D, folate, iodine, selenium, and long-chain *n*-3 polyunsaturated fatty acids (omega-3 fatty acids).

Several limitations are noted in this case–control study. Our data set was drawn from patients who delivered at the clinic; subsequently there is selection bias due to the missing home births. We were unable to collect diet and morbidity data during pregnancy. Given that participants were months to years out of pregnancy, the physiologic and socioeconomic environment of our study participants might have changed, impacting our findings. Lack of inclusion of rural study participants also limits the applicability of the study outside an urban setting similar to Cap-Haïtien, Haiti. Erratic phone services limited reaching potential study participants. In addition, the study had a small sample size and thus lacked study power; future studies with larger sample sizes would be beneficial.

Our study offers preliminary insight into nutrition-related factors influencing birthweight outcomes and the prevalence of hypertension among women in a low-resource setting. More research is needed to examine the multifaceted problem of LBW and potential types of nutrition and interventions to improve birth outcomes. These studies will also require consideration of births that occur outside the healthcare system, such as in rural villages. Identifying the most effective nutritional intervention to reduce LBW is even more critical, with recent findings from the International Lipid-Based Nutrient Supplement trials showing only limited effects on reducing birth outcomes.^25^ More research is needed to examine the effects of improving dietary diversity more broadly, and the use of affordable animal source foods such as eggs as sustainable alternatives to addressing LBW.

## Abbreviations Used

FAO: Food and Agriculture Organization
IRB: Institutional Review Board
LBW: low birth weight
NBW: normal birth weight
PR: prevalence ratio
WDDS: Women's Dietary Diversity Score
